# Problematic Use of Cannabis in Cotonou: Profile of Some Subjects Received in the Laboratory between 2016 and 2021

**DOI:** 10.1155/2022/9702766

**Published:** 2022-10-28

**Authors:** Ferdinand M. Adounkpe, Luc Behanzin, Bienvenu S. Adehan, Marc Medehouenou, Ornella Daoudou, Assad Bio-Sya, Odile Kougblenou, Arnaud Agbanlinsou, Clément Agbangla

**Affiliations:** ^1^National Laboratory of Narcotics and Toxicology (NLST)/Beninese Center for Scientific Research and Innovation (CBRSI)/University of Abomey-Calavi, Abomey-Calavi, Benin; ^2^National School for Training of Senior Technicians in Public Health and Epidemiological Surveillance, (ENATSE), University of Parakou, Parakou, Benin; ^3^Population Health and Optimal Health Practices Axis, Research Center of the CHU de Québec – Laval University, Quebec, Canada; ^4^Institut National de Recherches Agricoles du Benin (INRAB) Cotonou, University of Abomey-Calavi, Abomey-Calavi, Benin; ^5^Research Laboratory in Applied Biology, Polytechnic School of Abomey-Calavi, University of Abomey-Calavi, Abomey-Calavi, Benin; ^6^Laboratory of Sociology and Applied Medical Anthropology (LAMA), University of Abomey-Calavi, Abomey-Calavi, Benin; ^7^Laboratory of Histology, Reproductive Biology, Cytogenetics and Medical Genetics (LHBRCGM), University of Abomey-Calavi, Abomey-Calavi, Benin; ^8^Laboratory of Genetic and Biotechnology (LGB), University of Abomey-Calavi, Abomey-Calavi, Benin

## Abstract

**Aims:**

The frequent use of cannabis by certain social strata often induces behavioral changes whose severity deserves to be evaluated. This study aims to describe the profile of some subjects in a situation of cannabis dependence received at the National Laboratory of Narcotics and Toxicology over the period from January 2016 to December 2021. *Methodology*. The approach of direct interviews with the respondents using a semistructured questionnaire made it possible to collect their sociodemographic characteristics, their experiences, and their reference trajectories.

**Results:**

A total of 48 patients, all single, with an average age of 18.13 ± 0.48 years, the majority of whom were male (77.08%) and of Beninese nationality (85.42%), were enrolled. They were pupils (58.34%), students (27.08%), and workers (14.58%). With a prevalence of consumption of 89.58%, the first experimentation of cannabis was done by imitation (83.3%), among friends (81.2%), and in schools (64.58%). The reasons for use include the search for thrills (29.20%) and the improvement of sexual energy performance (27.1%). Subjects between 64.58 and 79.17% reported having received complaints about their behavior after regular consumption of at least 3 joints of cannabis.

**Conclusion:**

The knowledge of the typical profile of subjects in a situation of dependence ensures early detection of problematic uses of cannabis and offers the opportunity to intervene a little earlier in front of this phenomenon.

## 1. Introduction

*Cannabis sativa* is a genus of the Cannabaceae family known as Indian hemp. It is a plant species used for its neuropharmacological effects which are mainly due to its main psychoactive ingredient which is delta-9-tetrahydrocannabinol, commonly called THC [1]. Classified as a narcotic, *C. sativa* comes in 3 essential forms: the herb (dried leaves, stems, and flowering tops, marijuana), the resin (the “hashish”), and the oil (more concentrated in active ingredient). Herb and hashish are generally smoked in the form of a “joint” (i.e., with tobacco in the form of a rolled cigarette). Oil is usually consumed through a pipe. More marginally, cannabis can also be ingested and incorporated into food preparations (space cakes) or drunk (infusions) [1].

The World Drug Report 2019 indicates that the cultivation and consumption of cannabis has been on the rise on all continents since 2010 [2]. Cannabis is the number one illicit substance used by adolescents [3] and whose early experimentation is constantly progressing in Europe [4].

Among the youngest, early experimentation is mainly among boys, while later on, the differences in levels between boys and girls are clearly reduced [5]. Cannabis has euphoric, disinhibiting, and relaxing effects. From a health point of view, its use can be accompanied by acute psychiatric disorders but also, in the case of chronic use, by anxiety and depressive disorders such as psychotic disorders, without necessarily presuming a causal link [1]. Cannabis uses are remained at a high level with prevalences varying between countries. In 2018, cannabis herb seizures reached a new historical level with over 30 tons (+50% compared to 2017) [6]. The growing importance of the herb market in France is corroborated by the scale of plant confiscations, of which more than two hundred tons were seized on the territory in 2018 [7]. With more than 4 million current users in the population aged 11 to 75 years, including more than 2 million regular users, France is one of the countries with the highest cannabis use [8–10]. Nearly half of young adults (15–34 years) report having used cannabis at some time (45.1%), the European average being 32.5%, 17.5% have used it in the past-year (vs. 12.4% in Europe), and 9.8% in the past 30 days (vs. 6.6% in Europe). The most recent epidemiological data confirm that cannabis use is most often abandoned during the transition to adulthood [11]. In other words, although frequent cannabis use increases the risk of future harm, many frequent users do not report any disorders associated with their cannabis use. These findings suggest that not all cannabis use is problematic [11–13]. Problematic use thus falls between risky use and abuse. It is used that begins to cause psychosocial disruption. Problematic use refers to patterns of use that have been defined as risky in empirical studies. Individuals diagnosed as being in a situation of abuse or dependence are most certainly in a situation of problematic cannabis use, but not all problematic uses refer to a situation of dependence. Research studies that refer to the concept of problematic use [14–18] define it as use that leads to negative health or social consequences for the individual or for society. According to Davis et al., problematic use is defined as a consumption behavior likely to lead to harm (social, physical, financial, and professional), or a situation of abuse or dependence in a particular population [19]. Problematic cannabis use can therefore be defined as use likely to result in negative individual or social consequences [12].

Cannabis is the favorite drug in Africa with record seizures of several hundred tons in Nigeria, Morocco, Congo, and Sudan [4]. Cannabis is the main drug in circulation in Benin with a hundred arrests for more than ten tons seized in Cotonou over the period of 2013 and 2017 [20]. Cannabis continues to be consumed in Benin despite all the actions implemented by local authorities. Cannabis use has been identified as a factor associated with shisha smoking among students in Benin [21]. The availability and at a lower cost of Cannabis in Cotonou is a factor favoring its early use among young people, especially adolescents [22] which represent more than 43.5% of the total Beninese population in 2016 [23]. The absence in Benin of an operational national data collection system makes it difficult to correctly estimate the extent of drug addiction [24]. Cannabis use trends in Benin in high schools and colleges in Cotonou which are similar to those reported by the 2011 report of the ESPAD survey in France where more boys than girls used cannabis before the age of 14 years [25].

Identifying and assessing the problematic use of *C. sativa* in the young population is a public health issue recognized by the WHO, given the health and social consequences that such use can have, particularly in the case of early experimentation. However, primary care workers (general practitioners, school nurses, etc.), who are in the front line of perceiving these addictive disorders, often confess to being at a loss as to the strategies to adopt when dealing with at-risk users. For their part, at-risk users rarely take the initiative to discuss this issue in hospital consultations. Although the demand for treatment has been increasing in recent years, the proportion of cannabis users seeking treatment remains low, even among those who are considered dependent. Depending on the study and the country, the proportion of people seeking treatment is around one-third of those diagnosed as dependent on cannabis, with general practitioners being the most frequently approached [26–28]. One of the factors explaining this level of demand for assistance seems to be the lack of opportunities for the early management of cases of problematic cannabis use: primary healthcare professionals seem insufficiently equipped to deal with this type of request, often feeling that they have only partial knowledge of the feasibility and effectiveness of different treatment methods.

Early experimentation with psychoactive substances among young people presents a high risk of problematic use of cannabis, which can cause significant health and social damage. To our knowledge, in Benin, studies on cannabis use have not yet focused on the issues of early identification of problematic use. This is why it is important not only to raise awareness among frontline health workers about the risks of cannabis use but also to work on the early detection of problematic use of this product. The present study, initiated in the laboratory to construct a typical profile of users at risk of cannabis dependence, is a promising first experience in Cotonou.

## 2. Materials and Methods

### 2.1. Site, Type, Inclusion Criteria, and the Objective of the Study

This was a cross-sectional study carried out at the National Laboratory of Narcotics and Toxicology (LNST) in Cotonou which took into account subjects received at the laboratory for 5 years, from January 2016 to December 2021. The study population was made up of individuals who were known to use cannabis, essentially pupils, students, and workers whose use of cannabis had worried their parents and had required consultations with several doctors in Benin and elsewhere before their arrival at the laboratory. Only subjects taken to the laboratory by their parents to control their cannabis use were included in this study. All of them were in treatment with general practitioners or psychologists in Benin, Europe, and the USA with the aim of reducing or even abandoning their use of cannabis.

They were all accompanied to the laboratory by their parents or guardians, whether willingly or not, and most of them were not informed of their parents' or guardians' approach. The aim is to check their cannabis consumption after the numerous expensive treatments they have been subjected to for months or even years. When they arrive at the laboratory, they are taken in charge and put in confidence then informed of the reasons of their visit to the laboratory. They are invited to collaborate to reassure the attendants by answering the questions and by agreeing to give their urine for a drug identification control. The semiadministered questionnaire collects information on their sociodemographic characteristics (age, sex, occupation, marital status, nationality, attitude of the subject when he/she arrives at the laboratory, places and habits of cannabis use, medical and behavioral history, and consent to come to the laboratory) and during the first reception interview at the laboratory (reason for coming to the laboratory, most commonly used products, age and place of first experimentation, companions of use, reasons, date of last use before visiting the laboratory, place of supply and average monthly expenditure for buying cannabis, other means of supply, how long do you go without using cannabis, sensations after use, number of joints at each intake, complaints against you, loss of friends, worries with the family, have you tried to stop, do you want to stop, do you want help, do you accept to be followed from now on, have you ever consulted a doctor, have you ever followed a treatment, and do you want to give a little urine to check if you use cannabis or not).

The anonymity and confidentiality of the data collected were in accordance with the ethical principles applicable to medical research on human subjects contained in the Declaration of the World Medical Association of Helsinki [29].

The data collected and processed during this study contributed to the achievement of its objective, which was to determine the typical profile of users at risk of Cannabis dependence received at the LNST between 2016 and 2021.

### 2.2. Statistical Analysis

Data processing and analysis were carried out with the EPI Info software version 7.1.3.3. The quantitative variables were described by the parameters of central tendency and dispersion. The qualitative variables were described by the proportions with their confidence intervals if necessary. Pearson's Chi-square and associated tests of homogeneity were used to compare proportions in order to identify associated factors. For the entire study, the significance level of 5% was chosen for statistical interpretations.

## 3. Results

A total of 48 subjects were received at the Laboratory between 2016 and 2021 for their problematic use of Cannabis. The following results helped to construct a typical profile of these users in Cotonou.

### 3.1. Sociodemographic Characteristics of Subjects

The analysis of the sociodemographic characteristics of the cannabis users was carried out in consideration of the variables age, sex, occupation, nationality, and marital status (Table 1). From the analysis of the above table, it appears that all the subjects received in the laboratory were single, pupils (58.33%), students (27.08%), or workers (14.58%). The majority were male (77.08%) and of Beninese nationality (85.42%). The average age was 18.13 ± 0.48 years, and most were under 18 years of age (52.08%).

### 3.2. Subjects' Attitudes

**Figure 1:**
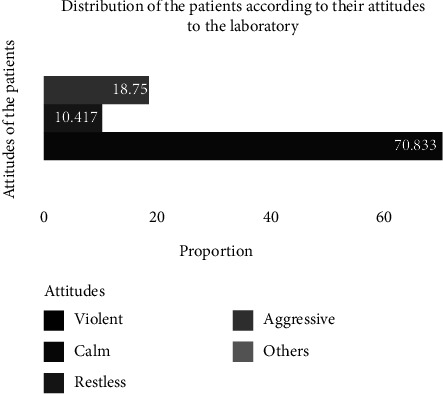
Distribution of the subjects according to their attitudes.

Figure 1 reports the different attitudes of the subjects when they arrived at the laboratory.

On arrival at the laboratory, the majority of patients had a calm attitude (70.83%) and some were either aggressive (18.75%) or agitated (10.42%).

### 3.3. Location of Cannabis Use

**Figure 2:**
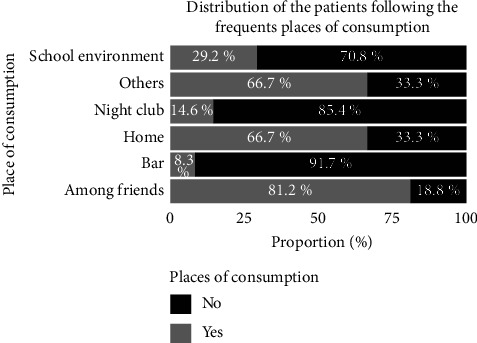
Distribution of the subjects according to locations of cannabis consumption.

Figure 2 reports the locations of cannabis use reported by the subjects received in the laboratory.

The majority of cannabis users used cannabis in schools (66.67%).

### 3.4. Beginning of Use

The analysis of Table 2 reveals that the majority of the patients received have an onset of cannabis use that goes back to less than one year (54.17%).

### 3.5. History of Violence

**Figure 3:**
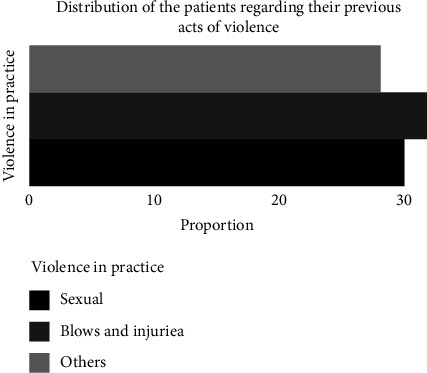
Distribution of the subjects according to their histories of violence.

Figure 3 reports the different histories of violence of the subjects received in the laboratory.

The history of violence most practiced by the cannabis users received at the laboratory is assault and battery (35.41%), sexual violence (33.33%), and other types of violence (31.25%).

### 3.6. Academic Concerns

**Figure 4:**
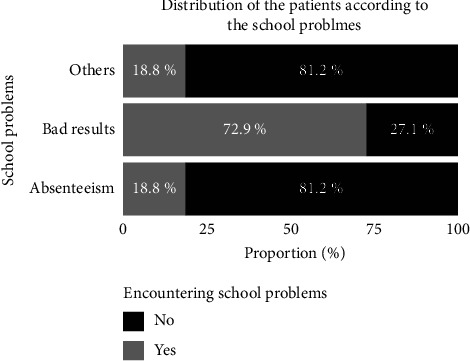
Distribution of the subjects according to their academics worries.

Figure 4 reports the different worries declared by the subjects received at the laboratory. From Figure 4, it can be seen that the most common academic problems encountered by the subjects received in the laboratory are related to poor results (72.9%).

### 3.7. Previous Treatment and Duration of Treatment

**Figure 5:**
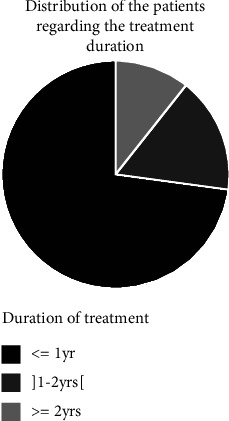
Distribution of patients according to the duration of their treatment.

Among the cannabis users interviewed, some had previously received detoxification treatments.  Figure 5 presents a distribution of patients according to the duration of their treatment.

Figure 5 shows that more than half of the patients (72.92%) had received treatment for less than one year, less than a quarter (16.67%) had received treatment for 1 to 2 years, and a small proportion (10.42%) had received treatment for more than two years. It should be noted that the category of those who have already received treatment is the least represented among the subjects (35.42%). In fact, the majority (64.58%) had never followed a previous detoxification treatment before their arrival at the laboratory.

### 3.8. Places of First Experimentation and Frequent Use

**Figure 6:**
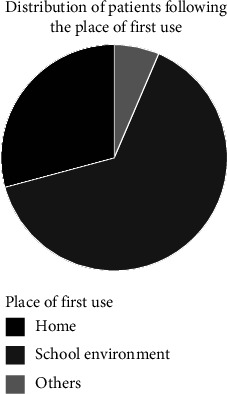
Distribution of the patients according to the place of the first experimentation.

**Figure 7:**
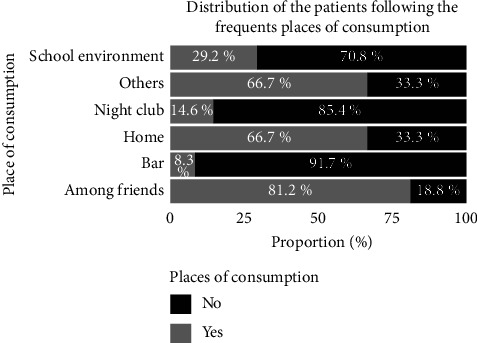
Distribution of the patients according to the place of frequent consumption.

Figures 6 and 7 present, respectively, the distribution of the patients according to the place of the first experimentation and the place of frequent consumption.

For more than half of the subjects (64.58%), the first experimentation of cannabis was conductedin a school environment (Figure 6) while the most frequent places of consumption declared were as follows: “at friends' houses” (81.2%), and home and other places (66.7%).

### 3.9. Consumption Companions and place of Procuration

**Figure 8:**
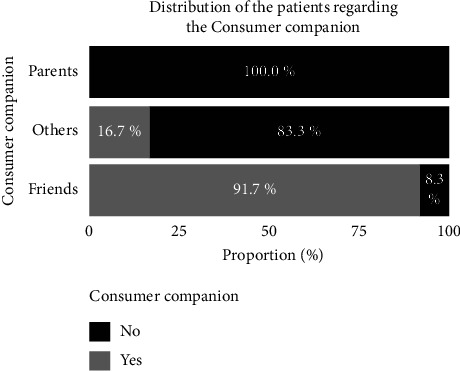
Distribution of the subjects according to the companions with whom they frequently consume cannabis.

**Figure 9:**
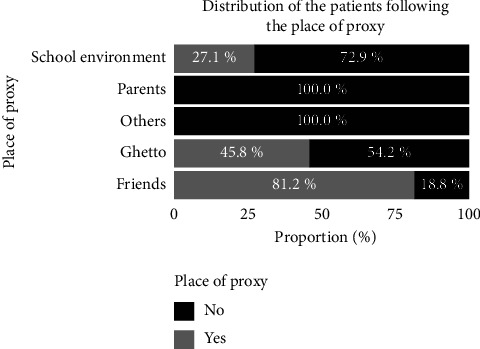
Distribution of the subjects according to the place of procurement.

Figure 8 presents the distribution of the subjects according to the companions with whom they frequently consume cannabis. Figure 8 reveals that friends represent the most frequent drinking companions of patients (91.7%).  Figure 9 presents a distribution of the subjects according to the place of procurement. Most of the subjects surveyed said they obtained their supplies from friends (81.2%), in the ghettos (45.8%), and in schools (27.1%).

### 3.10. Frequently Consumed Products and Monthly Expenses for Their Supply

89.58% of the subjects monitored use of cannabis more frequently compared to 10.42 who do not use it. While most of the subjects spend between 5.000 and 10.000 CFA francs per month (43.750%) or less than 5.000 CFA francs (41.67%), the minority spend more than 10.000 CFA francs (14.58%).

### 3.11. Age of First Use

On average, the cannabis users interviewed were 14 years old when they first used cannabis. Half of them admit to having used cannabis for the first time at 13 years old. The maximum value recorded is 18 years.

### 3.12. Reason for Use

We note that the majority of the subjects have as reason of consumption the imitation (83.3%), while the weakest reason of consumption is the intellectual performance (10.4%). 27.1% of the subjects have as reason of consumption the sexual performance and the energetic performance, while the 29.2% of the subjects have the intellectual performance.

## 4. Discussion

The particularity of this study lies in the fact that only 48 subjects are included whose parents have freely taken them to the laboratory to monitor their use of cannabis. This approach is highly responsible, because it is very difficult for people to come freely to consult on health situations as sensitive as drug use. This initiative deserves to be congratulated and strongly encouraged. Very few parents make this laudable decision, which explains the small sample size of this study. Despite the low number of subjects, this is already an opportunity to communicate on this phenomenon in the hope that many more parents will find themselves in confidence and commit to a process of early detection of problematic cannabis use.

### 4.1. Sociodemographic Characteristics of the Subjects

Studies on cannabis use have often set aside individual characteristics to emphasize different uses and practices. Following Simmat-Durant's logic, since the 2000s, researchers have tended to take into account the gendered dimensions of consumption, and it was at this time that the first studies on female users were published [30]. In Beninese society, girls are not as rebellious as boys, who do not hesitate to take more risks, sometimes defying parental authority. It is therefore young men who are more concerned about problematic cannabis use in Benin.

Although drug use is still perceived today as a more masculine practice [30–33] and mainly affects young people, especially males [9], women are also involved in these practices, which partly explains the low percentage of female subjects involved in this study. This result is supported by the study of Beck et al., who estimate that in the general population, men are still more likely to use cannabis regardless of the frequency of use, from experimentation to daily use, but the gap tends to decrease in recent years [11]. The sex ratio among young adults reporting past-year cannabis use is more than two men to one woman in France. For example, this ratio varies from just over six men to one woman in Portugal to less than one in Norway. However, the sex ratio tends to increase with the frequency of use [34].

Subjects included in the study are very young. This result is in agreement with the global situation in the countries in that the first experimentation of cannabis is generally conducted in adolescence, in middle school, or more generally in high school with peers.

Most research studies show that cannabis is the first illicit substance used at a very early age in the school or learning environment. The average age of onset of cannabis use in our study is similar to that observed between 2014 and 2021 in several studies of young adolescents in the main countries of the European Union: experimentation with cannabis during the junior high school years does not really begin until the 4th grade at the age of 15 to 18 when one in ten students had reported having smoked cannabis at least once [33,35–37].

As age is often associated with education level, individuals with lower education levels are over-represented in problematic cannabis use. It is known that drug users are often characterized by lower levels of education, as where the upper classes are experimenters, they rarely switch to problematic use [33].

Problem drug use peaks between the ages of 15 and 25. Beyond the age of 25, the proportion of current users gradually declines [34].

The most important sociodemographic variable in problematic use is age. Young people aged 15 to 24 are the most likely to have problematic cannabis use, so it is important to look at them in more detail. This situation leads the Health Barometer to reconsider these two essential questions: why are the youngest categories more likely to have a problematic use than the older categories? Are young people really a population at risk? [33]. The debate remains open.

Participants were pupils, students, or workers. According to Barometer Santé 2014, the socioprofessional category most represented in problematic uses are employees, workers, retirees, and young learners (pupils, students, apprentices). In other words, the more intensive the use, the more problematic the individuals are; this may explain their categorization between nonproblematic and problematic users [33].

It should be noted that all patients were accompanied when they arrived at the laboratory. This would indicate the social support that these young cannabis users have. Young users in a situation of cannabis dependence are a real headache for families. Parents are willing to sacrifice everything to save them from this practice that continuously ruins their lives. Some much older parents feel very painfully the psychological burden and often exorbitant cost of treatment for their children.

### 4.2. Attitudes of Cannabis Users

The attitude of the subjects who were not calm when they arrived at the laboratory could be explained by the fact of surprise. They had not been warned by their parents and probably some were still under the effect of products they had already taken. This could also be simply due to fear. Indeed, according to Dumais et al., cannabis use could affect the ability to control aggressive impulses, induce paranoid feelings, anxiety, and panic. In addition, abstinence and withdrawal could contribute to irritability and lead to risks of emotional outbursts.

### 4.3. Location of Cannabis Use

Results are in agreement with the data observed in the literature, the vast majority of learners have their first experience of cannabis use in middle and/or high school [3,13]. Experimentation among older learners in the general population has been going on for a long time and is more common among men than women, who have continued to use it since adolescence [38].

### 4.4. Violence

The most frequent violent acts experienced by cannabis users are physical (assault and battery).

Adolescents and young adults who regularly use cannabis are 2.8 times more likely to be involved in physical violence, according to a meta-analysis by the Research Centre of the University Institute of Mental Health of Montreal, published in the American Journal of Psychiatry.

### 4.5. School Worries

The school worries encountered by the subjects are essentially poor results and absenteeism. A question frequently raised in the literature in relation to academic concerns is the relationship between this phenomenon and other problematic behaviors in youth, particularly alcohol and drug use and abuse.

Youths with a substance abuse profile are a population of so-called at-risk youths, most of whom perform poorly in school. According to Eggert et al., these youth are considered to be at high risk to society because their future may involve chronic failure, nonproductive adulthood, health problems, crime, alcoholism, drug abuse, poverty, unemployment, and welfare-related difficulties. Hence, the challenge for school and health professionals is to prevent these at-risk youth from falling into school dropout and substance abuse.

Even when these youth drop out of school, they have difficulty maintaining employment when they find it. Indeed, according to Palle, there is a link between APS use and negative effects in the workplace, the latter of which can result in absenteeism, presenteeism, decreased performance and thus impacting the bottom line of businesses [39].

### 4.6. Previous Treatment, Duration of Treatment, and Reasons for Use

The participants have already been received in treatment with general practitioners or psychologists before their arrival at the laboratory. The literature also reports that the use of cannabis was motivated by various reasons and led an important part of the consumers (40–50%) to come into contact with structures or health professionals for psychological, psychiatric, and preventive care in addictionology [39,40].

The limitations of this study could be located at three levels: (i) first, in most cases, the subjects received in the laboratory were taken there without their consent, and the reception interview proved to be very difficult, some being silent and others much more violent. Many subjects were reluctant to answer questions about their cannabis use. A larger number of subjects responded after reassurance and explanation of the purpose of their visit to the laboratory by the laboratory officer, which had the direct consequence of unnecessarily delaying the interviews; (ii) second, only 48 subjects are involved in this study, and this number is not high and may not be more representative of the population of problematic users in Cotonou. The limited recruitment in Cotonou exposed sociodemographic differences compared to subjects in the rest of Benin. In addition, the sex ratio of our sample showed a high proportion of men, which could imply a selection bias; (iii) finally, our study was based on a semiadministered questionnaire. The collection of data by this method exposed risk of bias in the influence of the answers and of classification by over or underestimation of the information. In addition, there was the risk of the subjects' reserve concerning the declarations about their drug use and the fear of possible consequences. Despite these limitations, this study has the advantage of drawing attention to the problematic use of cannabis in Cotonou. It also allows parents faced with this situation to have the courage to consult their children in a situation of Cannabis addiction.

## 5. Conclusions

The need for early detection of problematic cannabis use was again confirmed by this study, which showed that the majority of *C. sativa* users starts their cannabis use very early and modify it as they go along, increasing the risk of risky use and dependence. Addressing this issue during a primary care consultation can help identify problematic use that is taking hold, and help the user to reflect and become aware of his or her use, which is a prerequisite for any change in behavior. It seems useful to insist very early on, first of all, on the importance of opening a dialogue with the user about his or her cannabis use, then of researching and evaluating the serious factors associated with this consumption. It is necessary to provide assistance to the user in a situation of problematic cannabis use by offering him or her opportunities for care available today or by directing him or her to a specialized monitoring and treatment center. In the laboratory, the dialogue with the user was based on a questionnaire, which made it possible to reveal the characteristics of the use of the subjects surveyed in order to establish a typical profile of the user in a problematic use situation in Cotonou, Benin. It is therefore important not only to raise awareness among frontline health workers about the risks of problematic cannabis use among young people but also to encourage them to work on the early identification of risky cannabis use.

## Figures and Tables

**Table 1:** Sociodemographic characteristics of subjects.

Variables	Terms	Effectives (frequency) (%)
Sex	Male	37 *(77.08)*
	Feminine	11 *(22.92)*

Age (18.13 ± 0.48 years)^*∗*^	<18	25 *(52.08)*
	[18–20[	8 *(16.67)*
	[20–22[	6 *(12.50)*
	≥22	9 *(18.75)*

Occupation	Pupil	28 *(58.33)*
	Student	13 *(27.08)*
	Worker	7 *(14.58)*

Nationality	Beninese	41 *(85.42)*
	Other	7 *(14.58)*

^*∗*^mean ± standard error.

**Table 2:** Period of beginning of cannabis use.

Beginning of cannabis consumption (years)	Effectives (%)
≤1	26 (*54.17*)
]1–2 [	5 (*10.42*)
≥2	17 (*35.42*)

## Data Availability

Datasets generated and/or analyzed during the current study are available from the corresponding author upon reasonable request.
